# Classical and Non-classical Fibrosis Phenotypes Are Revealed by Lung and Cardiac Like Microvascular Tissues On-Chip

**DOI:** 10.3389/fphys.2021.735915

**Published:** 2021-10-06

**Authors:** Akinola Akinbote, Violeta Beltran-Sastre, Marta Cherubini, Roberta Visone, Cynthia Hajal, Defne Cobanoglu, Kristina Haase

**Affiliations:** ^1^European Molecular Biology Laboratory, Barcelona, Spain; ^2^Heidelberg University, Faculty of Biosciences, Heidelberg, Germany; ^3^Politecnico di Milano, Department of Electronics, Information, and Bioengineering, Milan Italy; ^4^Massachusetts Institute of Technology, Department of Mechanical Engineering, Cambridge, MA, United States

**Keywords:** fibrosis on-chip, cardiac fibrosis, pulmonary fibrosis, microvasculature, microfluidics, ECM remodeling, TGF-β, matrix metalloproteases

## Abstract

Fibrosis, a hallmark of many cardiac and pulmonary diseases, is characterized by excess deposition of extracellular matrix proteins and increased tissue stiffness. This serious pathologic condition is thought to stem majorly from local stromal cell activation. Most studies have focused on the role of fibroblasts; however, the endothelium has been implicated in fibrosis through direct and indirect contributions. Here, we present a 3D vascular model to investigate vessel-stroma crosstalk in normal conditions and following induced fibrosis. Human-induced pluripotent stem cell-derived endothelial cells (hiPSC-ECs) are co-cultured with (and without) primary human cardiac and lung fibroblasts (LFs) in a microfluidic device to generate perfusable microvasculature in cardiac- and pulmonary-like microenvironments. Endothelial barrier function, vascular morphology, and matrix properties (stiffness and diffusivity) are differentially impacted by the presence of stromal cells. These vessels (with and without stromal cells) express inflammatory cytokines, which could induce a wound-healing state. Further treatment with transforming growth factor-β (TGF-β) induced varied fibrotic phenotypes on-chip, with LFs resulting in increased stiffness, lower MMP activity, and increased smooth muscle actin expression. Taken together, our work demonstrates the strong impact of stromal-endothelial interactions on vessel formation and extravascular matrix regulation. The role of TGF-β is shown to affect co-cultured microvessels differentially and has a severe negative impact on the endothelium without stromal cell support. Our human 3D *in vitro* model has the potential to examine anti-fibrotic therapies on patient-specific hiPSCs in the future.

## Graphical Abstract

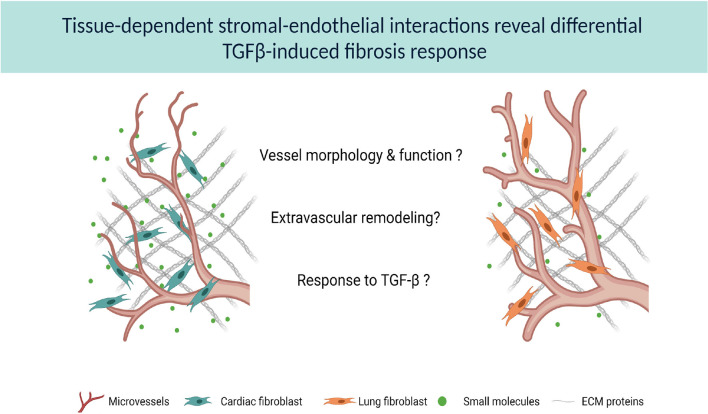

## Introduction

Organ fibrosis is responsible for a third of all fatalities globally and is a clinical hallmark of many cardiac and pulmonary diseases (Zeisberg and Kalluri, 2013). Fibrosis is characterized by excess deposition and remodeling of extracellular matrix (ECM) proteins, immune cell activation, and

increased tissue stiffness, wherein a continued activation of fibroblasts results in a myofibroblast phenotype (Hinz et al., 2001; Wynn and Ramalingam, 2012). In both cardiac and pulmonary tissues, fibrosis can be divided up into two stages—characterized by matrix remodeling, inflammation, initial myofibroblast activation during the early stages; and increased matrix accumulation (scarring), presence of macrophages, and continued fibroblast activation during the later stages (Murtha et al., 2017). Myofibroblasts are implicated in excessive deposition of ECM proteins, are hyperproliferative, express alpha-smooth muscle actin (αSMA), and are more contractile than the native stromal cell population. As a result, there is a subsequent build-up of ECM proteins, such as collagen, which in turn increases tissue stiffness and activates more fibroblasts toward a myofibroblast phenotype—resulting in a deleterious positive feedback loop. While ECM deposition is an essential part of the wound healing cascade, if left unchecked, it can lead to pathological fibrosis. This compromises the structure and function of the native tissue, eventually resulting in organ failure (Anversa et al., 1991).

Various etiologies of cardiac and pulmonary diseases, such as idiopathic pulmonary fibrosis and ischemic heart disease, involve fibrotic tissue remodeling prior to the clinical manifestation of end-stage organ failure (Wynn and Ramalingam, 2012). While several factors such as microenvironmental stiffness, reactive oxygen species, growth factors, and cytokines, have been implicated in both cardiac and pulmonary fibrosis, transforming growth factor-β (TGF-β) has been identified as a central actor (Wynn and Ramalingam, 2012; Koliaraki et al., 2020; Wang et al., 2020, 2021). Continued secretion of TGF-β results in increased proliferation and activation of myofibroblasts resulting in dysfunctional ECM remodeling. Despite the many studies on fibrosis using *in vitro* and *in vivo* models, we are still limited in our understanding of disease progression in regards to the fibrotic response. This limitation has been attributed, in part, to inadequate humanized models which often fail to capture complex pathophysiology and predict drug interactions; animal models (*in vivo)* do not account for species-dependent differences while 2D models (*in vitro*) lack the complexity to model human tissue. As such, there has been a move toward more complex *in vitro* 3D models such as microfluidic (on-chip) and organoid systems.

*In vitro* models of cardiac and pulmonary fibrosis have mainly focused on stromal-parenchyma interactions and have largely excluded the role of the endothelium in modulating the fibrotic response (Alsafadi et al., 2017; Aghajanian et al., 2019; Mastikhina et al., 2020; Mejías et al., 2020; Sacchi et al., 2020; Wang et al., 2020). Endothelial cells (ECs), which line the vessels that pervade all tissues and actively participate in health and disease, have been implicated in pathological fibrosis, from its onset through its progression. It has been proposed that an endothelial mesenchymal transition (endoMT), vascular inflammatory response activation, endothelial senescence, and vessel rarefaction all contribute to fibrosis (Zeisberg et al., 2007a,b; Johnson and DiPietro, 2013; Pardali et al., 2017; Sun et al., 2020). While the role of the vasculature in fibrosis is becoming increasingly recognized, there is a need to characterize vascular phenotypic changes resultant from the onset of fibrosis. The use of functionally vascularized *in vitro* models that replicate hallmarks (adverse ECM remodeling, myofibroblast activation, and increased tissue stiffness) of cardiac and pulmonary fibrosis is yet to be achieved. Considering that both the heart and lung are highly vascularized, it is of critical importance to examine the role of vessels and stromal-endothelial crosstalk in tissue-specific fibrosis.

To address this unmet need, we employed a 3D perfusable microvascular model to first understand the contributions of stromal cells to microvascular and extravascular matrix remodeling. Next, we use this system to induce fibrosis using TGF-β in cardiac- and pulmonary-like vascular tissues in a controlled manner. By culturing human-induced pluripotent stem cell-derived endothelial cells (hiPSC-ECs) with (or without) human primary cardiac (CFs) or lung fibroblasts (LFs), we demonstrate the impact of these stromal cells on endothelial barrier function, microvascular morphology, and ECM properties. Subsequent antagonization of our system with TGF-β reveals the severe impact of a fibrotic phenotype on vascular stability, and the time and dose-dependent effects on microvascular tissues (μVTs) in lung vs cardiac-like microenvironments. Treatment resulted in differential effects on αSMA expression, matrix remodeling, MMP activity, and changes in vascular stability between the two microvascular tissue types. Using the hiPSC-EC derived microvasculature, we were able to investigate the crosstalk with the local stromal population and minimize tissue-dependent endothelial cell heterogeneity. Our findings suggest that lung and cardiac μVTs respond to TGF-β in a differential manner, with LFs contributing to the development of a classical fibrotic phenotype and cardiac fibroblasts a non-classical phenotype.

## Materials and Methods

### Cell Culture

Commercially available (and characterized) hiPSC-ECs were purchased from Cellular Dynamics (Fujifilm) and were cultured in endothelial media (VascuLife, Lifeline cell systems) with an additional 10 ml L-glutamine and 10% Fetal Bovine Serum on 30 μg/ml human fibronectin (Sigma) coated T-75 flasks. Primary normal human lung fibroblasts (NHLF) and primary normal human ventricular cardiac fibroblasts (NHCF-V) were purchased from Lonza. All cells were used between passages 5 and 7. NHLFs were cultured in Fibrolife media (Lifeline cell systems) on 50 μg/ml rat tail collagen I (Merck) coated T-75 flasks. NHCF-Vs were cultured in FibroLife supplemented with 10% FBS. All cells were cultured at 37∘C and 5% CO_2_, and a complete media change was conducted every other day. To culture the optimal cell numbers for device seeding, hiPSC-ECs were subdivided into 2 × T-150 flasks after the first passage and grown again to 80–90% confluency.

### Device Fabrication

As previously described (Haase et al., 2019), devices were fabricated using PDMS (SYLGARD^TM^ 184 Silicone Elastomer Kit, Dow). The elastomer and cross-linker were mixed in a 10:1 ratio, per manufacturer’s recommendations, degassed using a vacuum desiccator, and poured onto a fabricated mold, and degassed a second time. PDMS was then cured at 60∘C overnight and individual devices were cut, punched, and air-plasma bonded (Harrick systems) to clean glass slides. While hydrophilic, a 100 μg/ml Poly-D-Lysine (Sigma) coating was applied for >2 h before rinsing with sterile MilliQ water three times. These devices were then incubated at 60∘C overnight, reinstating hydrophobicity. Prior to cell seeding, all devices were sterilized under ultraviolet light for at least 30 min.

### Device Seeding and Formation of Microvessels

Fibrinogen derived from bovine plasma (Sigma) was reconstituted in phosphate-buffered saline (PBS) to a working concentration of 6 mg/ml before use. Thrombin (Sigma) was diluted to a 4 U/ml working solution in cold VascuLife medium. Endothelial cells and stromal cells were then dissociated and mixed with the appropriate volume of thrombin and fibrinogen solution to make up final concentrations of 6 million cells/ml and 1.2 million cells/ml, respectively, resulting in a 5:1 ratio, as previously described (Haase et al., 2019). To seed one device, an 18 μl cell + thrombin (final concentration of 2U) suspension was mixed with an equal volume of fibrinogen solution (final concentration of 3 mg/mL). Following insertion into the gel channel, the mixture was allowed to polymerize for 20–30 min at 37∘C in a humidified chamber. VascuLife media was supplemented with 50 ng/ml vascular endothelial growth factor A (VEGF; PeproTech) and was added to each media channel. The media was refreshed daily (150 μL) and cultured under static conditions.

### Transforming Growth Factor-β Treatments

On day 4, microvessels were treated with one of two TGF-β treatment regimens (low concentration/short-term and high-concentration/long-term), with daily media changes. The low concentration/short-term regimen consisted of a 5 ng/ml TGF-β supplemented growth media (replenished daily) until day 7 of culture. The high concentration/long-term regimen consisted of a 25 ng/ml TGF-β supplemented growth media (replenished daily) until day 11 of culture. For 2D experiments, lung and cardiac fibroblasts were seeded in 6-well plates at 100,000 cells per well using FibroLife growth medium (2% FBS). Cells were then treated with 0, 5, or 25 ng/ml TGF-β supplemented growth medium for 48 h, with complete daily media change.

### Cytokine Analysis

For 3D cytokine analysis, supernatants were pooled from *n* = 4 devices on day 5. We employed a human angiogenesis array (Abcam, ab134000) according to the manufacturer’s instructions. The relative expression of cytokines (measured by fluorescent intensity) was compared between all groups, corrected to the negative controls on each array, and normalized to the positive controls, using the monoculture as the reference array. For 2D cytokine collection, lung and cardiac fibroblasts were seeded in 6-well plates at 100,000 cells per well using FibroLife growth medium (2% FBS). Supernatants were collected after 48 h in culture. The cytokine profile was analyzed using the same human angiogenesis array (Abcam, ab134000). The relative expression of cytokines (measured by fluorescent intensity) was compared between all groups and normalized to the positive controls and negative controls. The blots were visualized using the Fusion FX Spectra (Vilber, France).

### Growth Factors

Exogenous VEGF (PeproTech, 100-20) was made up at a stock concentration of 100 μg/ml in 0.1% Bovine Serum Albumin (BSA) PBS and was supplemented in media at a concentration of 50 ng/ml. TGF-β1 (PeproTech, 100-21) was made up at a stock concentration of 50 μg/ml in 0.2% BSA 4 mM HCl and used at 5, 10, and 25 ng/mL, as indicated.

### MMP Expression

Supernatants were collected and pooled from *n* = 4 TGF-β treated devices on day 7, kept briefly on ice, then stored at −80∘C until use. Using two separate DuoSet ELISA KITs (R&D systems, DY901B and DY911), the concentration of MMP-1 and MMP-9 were determined for TGF-β treated conditions per the manufacturer’s instructions. Briefly, MMP concentrations were derived using measured absorbance values (with wavelength correction at 590 nm) compared to provided standards, accounting for the sample’s dilution factor. For MMP1 detection, samples were prepared in a 1:500 dilution with the reagent diluent. While for MMP9, samples were prepared in a 1:1 ratio. The growth media was used as a control, to account for any exogenous MMPs from the added FBS. Measurements were done in triplicate with lung and cardiac fibroblast only controls.

### Permeability Measurements

Microvessels were perfused on day 7 with 70 kDa FITC dextran (Merck) using a pressure gradient. Briefly, both media channels were emptied, then 40 μl of the fluorescent solute (100 μg/ml FITC dextran in vascular growth medium) was added to one media channel. Following perfusion through the microvessels, to halt convection, an additional 40 μl was added to the other media channel. After 1 min, time-lapse (3 × 3-min intervals) confocal *z*-stack images were acquired at a 5 μm step size and ≈20–25 slices. Analysis was done as previously described (Haase et al., 2019), using the equation below:


$$P\,\left(t\right)=\frac{A_{T}\,\left(I_{T_{f}}-I_{T_{0}}\right)}{p_{v}t(I_{V_{0}}-I_{T_{0})}}$$

Where *p_v_* is the vessel perimeter, *I* is the fluorescent intensity which is linearly related to the concentration of the fluorophore. *P*(*t*) is the approximated permeability *P* (cm/s) and *A_T_* is the extravascular tissue area.

### Vessel Morphology Quantification

The maximum projected images of the FITC-dextran channels at *t* = 0 were used to quantify the morphology of the microvascular networks. Briefly, a custom Fiji macro was generated to process the images as follows: projections of maximum intensity of the FITC channel in the *z*-direction, Gaussian filter smoothing (with 3 iterations with sigma values of 3, 2, and 2), followed by adaptive local thresholding using the Phansalkar method (*k* = 0.5, *r* = 0.75, at a radius of 150 pixels), binarization, and the removal of outliers of radius 3 pixels. The built-in *Analyze particles* and *2D skeletonize* plug-ins were employed (Supplementary Figure 2). The morphological quantifications were then normalized to the area of the fully perfused regions, as these measurements were performed on FITC-dextran channels.

### Extravascular Diffusivity

Small molecule diffusivity in the extravascular space was determined for each μVT condition using fluorescence recovery after photobleaching (FRAP), as previously described (Haase et al., 2020). After perfusion with a 70 kDa FITC-dextran into the microvessels, μVTs were incubated at 37∘C for ≥1 h, to allow for total diffusion throughout the hydrogel (in the intra- and extravascular space). Intra- and extravascular regions are still detectable, as shown by outlines in Figure 3A. Bleaching was performed in 30 μm diameter regions, with 30 s total bleach + recovery. Over 10 measurements were taken per device. Time-lapse imaging was performed to capture the bleaching and subsequent recovery of fluorescence in the extravascular regions. These images were then analyzed using a MATLAB FRAP analysis tool (Jönsson et al., 2008) to correlate the changes in fluorescence intensity with time (Figure 3B). The diffusion time was estimated using *L*^2^/D, where D is the diffusivity of the fluorescent particle and *L* is the maximum distance between blood vessels (in our case, the diameter of the bleached area; Dewhirst and Secomb, 2017).

**FIGURE 1:**
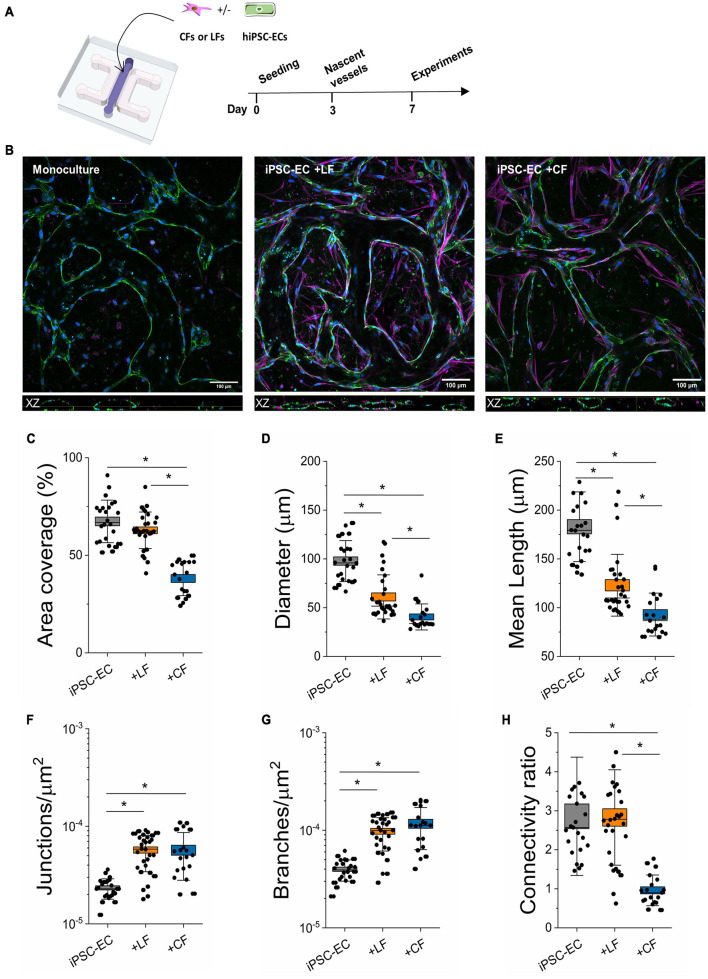
Stromal cells alter the morphology of hiPSC-EC derived microvessels. **(A)** Schematic of the on-chip PDMS device used to generate hiPSC-derived microvessels. Cells are encapsulated within a fibrin hydrogel in the central channel and form microvascular networks as early as day 3. **(B)** Representative confocal images of microvascular networks taken at 20x. L-R: Microvessels derived from hiPSC-EC monoculture, hiPSC-EC and lung fibroblast co-culture, and hiPSC-EC and cardiac fibroblast co-culture. Microvessels were stained for a known endothelial marker, CD31 (green), and were counterstained by phalloidin (magenta) and Dapi (blue). Top: XY plane of formed microvascular networks. Bottom: orthogonal (XZ-plane) images of microvessels showing open lumens. **(C–H)** Comparison of morphological parameters between mono- and co-cultures. Shown are data from 3 separate experiments with ≥10 devices per condition. Box plots demonstrate SD (outer whiskers) and SE (box edge). Significance is shown by *P < 0.05, using one-way ANOVA and a subsequent Tukey means comparison test.

**FIGURE 2:**
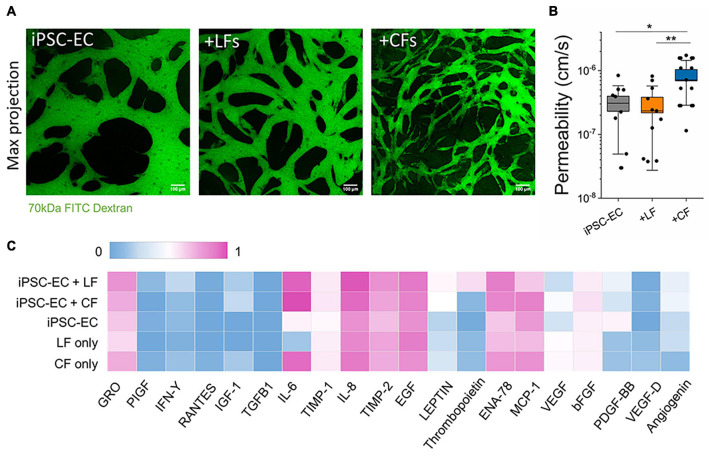
Endothelial barrier function is affected by stromal cells. **(A)** Confocal maximum projection images demonstrating microvessels perfused with 70 kDa FITC dextran (green). The scale bar is 100 μm. **(B)** Endothelial permeability to 70 kDa FITC dextran, measured at day 7. CF co-culture significantly decreases endothelial barrier function. Shown are data from 3 separate experiments. Box plots demonstrate SD (outer whiskers) and SE (box edge). Significance is shown by *P < 0.05, **P < 0.01 using a *t*-test to compare with mono-cultured and co-cultured vessels. **(C)** Cytokine profiling using an antibody array from supernatant collected from microvessels at day 5. The measured intensity of the expressed cytokines was normalized intensity to the positive and negative controls on the array.

**FIGURE 3:**
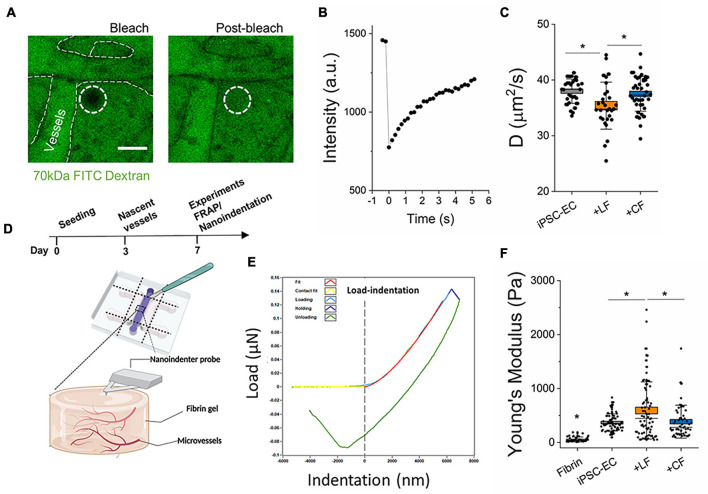
Stromal cells impact extravascular matrix properties. **(A)** Representative images of FRAP measurements in the extravascular regions performed on day 7. Images show bleached and post-bleached regions—as indicated by the dotted white circle. **(B)** Example of post-bleach recovery, as measured by fluorescent intensity over time. **(C)** Diffusivity measurements of the extravascular regions from the different microvessels. **(D)** Experiment timeline for FRAP and schematic of nanoindentation measurements. **(E)** A representative load-indentation graph of the gel-microvessel substrate. The vertical dotted line indicates the intersection of the contact point. **(F)** Measured effective Young’s modulus for Microvessels at day 7. Box plots demonstrate SD (outer whiskers) and SE (box edge). Significance is shown by *P < 0.05, using one-way ANOVA and a subsequent Tukey means comparison test.

### Immunofluorescence Staining

Fixation was performed using 4% paraformaldehyde for 20 min prior to washing with PBS and subsequent solubilization using 0.1% Triton-X (10 mins). Samples were then incubated in applicable blocking buffer, PBS + BSA + serum of the secondary antibody, for more than 1 h. Primary antibodies were diluted in wash buffer (0.5% BSA in PBS) and were added to the samples and incubated overnight at 4∘C. After overnight incubation, samples were washed with wash buffer and incubated with the appropriate secondary antibodies and counterstains (>2 h). Samples were rinsed with PBS and either imaged immediately or mounted on coverslips (for 2D samples) using Fluoromount-G (Invitrogen) and stored at 4∘C before imaging. To stain microvessels within a device, a pressure gradient was applied across the gel for all staining and wash steps.

### Nanoindenter Measurements

The effective Young’s Modulus of fibrin hydrogels was measured using the Chiaro Nanoindenter (Optics 11, Amsterdam, Netherlands). Nanoindentation measurements were done at room temperature using spherical probe tips with a mean radius of 28.5 ± 3.12 μm and an average stiffness of 0.026 ± 0.002 N/M, respectively. To access the gel/tissue in a device, a scalpel was gently used to cut away the top layer of PDMS with minimal gel agitation (Figure 3D). Prior to the probe insertion, the gel chamber was topped up with growth medium to ensure the gel remained fully hydrated during measurements. The probe was calibrated in media and then gently submerged into the liquid of the device containing the hydrogel. Measurements were performed with an indentation depth of 12 μm (∼2.4% of ∼500 μm thick hydrogel), using the manufacturer’s indentation control function in the adhesion mode. An approach speed of 50 μm/s was employed. The effective Young’s modulus was derived from load-indentation curves by fitting to the standard Hertz model and assuming a Poisson’s ratio of 0.5, using the manufacturer’s data analysis plug-in. On average, ∼16 measurements were analyzed per sample with an *n* ≥ 3.

### Statistics

Unless noted otherwise, one-way ANOVA was used to assess statistical significance across conditions at *P* < 0.05, and a *post-hoc* Tukey test was performed as a means comparison using OriginPro8. Data shown here are from *n* ≥ 3 devices with at least 2 measurements per device, except for the nanoindentation measurements (∼16 measurements per device) and unless otherwise specified.

### Western Blot

Gels were extracted using a scalpel to cut away the top layer of PDMS with minimal gel agitation. Each extract was directly transferred to 100 μl RIPA buffer on ice for tissue lysis. Samples were immediately frozen at −80∘C for at least 30 min prior to sonication. Extracts were homogenized on ice using a Bioruptor^>^ Sonication System with at least three freeze-sonicate-freeze cycles until the gel was fragmented. All samples were vortexed for 30 s and then centrifuged for 5 min at 4∘C at maximum speed. Protein concentrations were determined from the supernatant using the Pierce^TM^ BCA Protein Assay Kit according to the manufacturer’s protocols. 10 μg of measured protein in lysates were then added to 4x sample buffer (Laemmli buffer + DDT) and RIPA buffer to make a 50 μl solution. Samples were then heated at 95∘C for 5 min before gel loading in Mini-PROTEAN TGX Gels (BIO-RAD). Membranes were then blocked for 1 h on a rocker at RT in 5% nonfat dried milk powder in tris-buffered saline, 0.1% Tween-20 (TBST) after gel transfer. After blocking, antibodies for collagen-1 (Abcam, ab260043, 1:2000), α-SMA (Abcam, ab5694, 1:1000), and β-actin (Merck, A1978, 1:2000) were incubated at 4∘C overnight on a rocker plate. Antibody binding was quantified using horseradish peroxidase-conjugated secondary anti-mouse (Abcam, ab205719, 1: 10,000) or anti-rabbit (Abcam, ab205718, 1: 10,000) after 1-h incubation. The blots were visualized using the Fusion FX Spectra (Vilber, France). Protein expression was normalized to β-actin expression using ImageJ. 2D cultures were extracted using a cell scraper and lysed on ice with RIPA buffer, then immediately homogenized for 10 min prior to vortexing.

## Results

### Stromal Cells Impact Morphology of Human-Induced Pluripotent Stem Cell-Derived Microvasculature

Using our previous microfluidic design (Haase et al., 2019; Offeddu et al., 2019), hiPSC-ECs were cultured with (or without) human primary cardiac (CFs) or LFs to generate microvessels in tissue-like microenvironments (Figure 1A). Adapting our previously published protocol (Haase et al., 2019), cells were encapsulated in a fibrin gel, with an endothelial to stromal cell ratio of 5:1, in the middle chamber of a macro-scaled fluidic device. After several days in culture, hiPSC-ECs coalesce to form a well-connected vascular network with open lumens, as seen in confocal images for mono- and co-cultures (Figure 1B). Actin staining demonstrates stromal cell association with the endothelium in both co-cultures (Figure 1B and Supplementary Figure 1). Vascular morphology measurements were quantified in perfused regions of mono- and co-cultured vessels on day 7 of culture, revealing the strong influence of stromal cells in these networks (Figures 1C–H). Parameters including vessel (effective) diameter, area coverage, junction and branch densities, connectivity, and average branch length were all compared. Both lung and cardiac fibroblasts result in increased branching of the networks and reduced vessel diameters. Moreover, the average branch length is significantly reduced. Notably, in the case of the cardiac fibroblast co-cultures, the vessel area was also significantly reduced, and vessels appeared quite narrow (Figures 1C, 2 and Supplementary Figures 1, 2). Perfusion was demonstrated using fluorescently labeled dextran coursing through the microvessel lumen (Figure 2). Perfusion of cardiac co-cultures was difficult and did not always result in fully perfused networks across the vascular bed.

### Stromal Cells Affect Endothelial Barrier Properties

Perfusion with FITC-labeled 70 kDa dextran into the microvessels at day 7 allowed for measurements of vascular permeability, as previously described (Haase et al., 2019). By tracing the flux of the fluorescent solute from intra- to extra-vascular regions (see perfused vessels in Figure 2A) we determined that LFs did not affect barrier function; however, cardiac fibroblasts resulted in significantly reduced barrier function (increased permeability) compared to hiPSC-EC monoculture vessels (Figure 2B). The values reported for both mono-cultured and lung co-cultured microvessels are similar to those reported previously for hiPSC-EC vessels (Hajal et al., 2021) and similar to *in vivo* (non-human) measurements [summarized in Offeddu et al. (2019)]. We hypothesized that co-culture with stromal cells could contribute to an altered angiogenic profile. Therefore, we collected supernatants from microvessels (and fibroblasts seeded in fibrin gels alone) on day 5 post-seeding and performed a cytokine array. A semi-quantitative analysis demonstrated many similarities between the mono- and co-culture vessels (Figure 2C). There were pronounced differences in ENA-78 and PDGF-BB between mono- and co-cultures, and overall high levels of IL-6, IL-8, TIMP-1, and TIMP-2 in all microvessels; most of these cytokines are negligibly expressed in the culture medium alone (Supplementary Figure 6). Notedly, IL-6 is increased in both 3D cultured CFs alone and when co-cultured with hiPSC-ECs. A separate cytokine profile of 2D cultured CFs again showed an increased expression of inflammatory factors CFs in comparison to LFs, despite both being cultured in a low-serum (2% FBS) medium (Supplementary Figure 3A).

### Lung and Cardiac Fibroblasts Differentially Alter Extravascular Tissue

Fibroblasts are known to continually remodel the ECM *in vivo* and are implicated in tissue homeostasis and pathophysiology (Di Carlo and Peduto, 2018; Koliaraki et al., 2020; Buechler et al., 2021). However, less is known regarding their contribution to this process in the microvascular niche. Here, two approaches were employed to examine the impact of stromal cells on extravascular matrix remodeling. First, FRAP techniques, as previously described (Haase et al., 2020), were performed in the extravascular space to determine diffusivity. A 70 kDa FITC-dextran was perfused into the vessels, followed by incubation for several hours, to allow for total diffusion throughout the hydrogel (in the intra- and extravascular space). Vessel regions are still detectable in the device, as shown by outlines in Figure 3A. Time-lapse imaging was performed to capture the bleaching and subsequent recovery of fluorescence in the extravascular regions. These images were then analyzed using a MATLAB FRAP analysis tool (Jönsson et al., 2008) to correlate the changes in fluorescence intensity with time (Figure 3B). The diffusion time can be estimated using *L*^2^/D, where D is the diffusivity of the fluorescent particle and *L* is the maximum distance between blood vessels (Dewhirst and Secomb, 2017). With an *L* of 30 μm, mean diffusion times for 70 kDa size molecules for the cardiac-like, lung-like, and monoculture μVTs were 24.2 ± 0.32, 25.79 ± 0.59, and 23.76 ± 0.26 s, respectively. Microvessels in the LF co-cultures resulted in extravascular regions with significantly reduced diffusivity in comparison to mono-cultured hiPSC-EC vessels (Figure 3C). Surprisingly, the cardiac co-cultured microvessels did not result in any significant change in diffusivity compared with monocultured vessels. Second, we aimed to correlate our findings from FRAP measurements with the mechanical properties of the microvessel tissues. Nanoindentation experiments were performed on the microvessel tissues following 7 days in culture. By carefully cutting away the PDMS using a surgical blade, the nanoindenter was used to probe the tissue stiffness of the various microvessels (Figures 3D–F). As expected, hydrogels containing vessels are significantly stiffer than fibrin (cultured in devices until day 7) alone. However, more importantly, LF co-cultured vessels result in increased tissue stiffness, compared to both cardiac and mono-cultured hiPSC-EC vessels (Figure 3F) following 7 days of culture.

### Transforming Growth Factor-β Treatment Induces Fibroblast-Specific Matrix Remodeling

**FIGURE 4:**
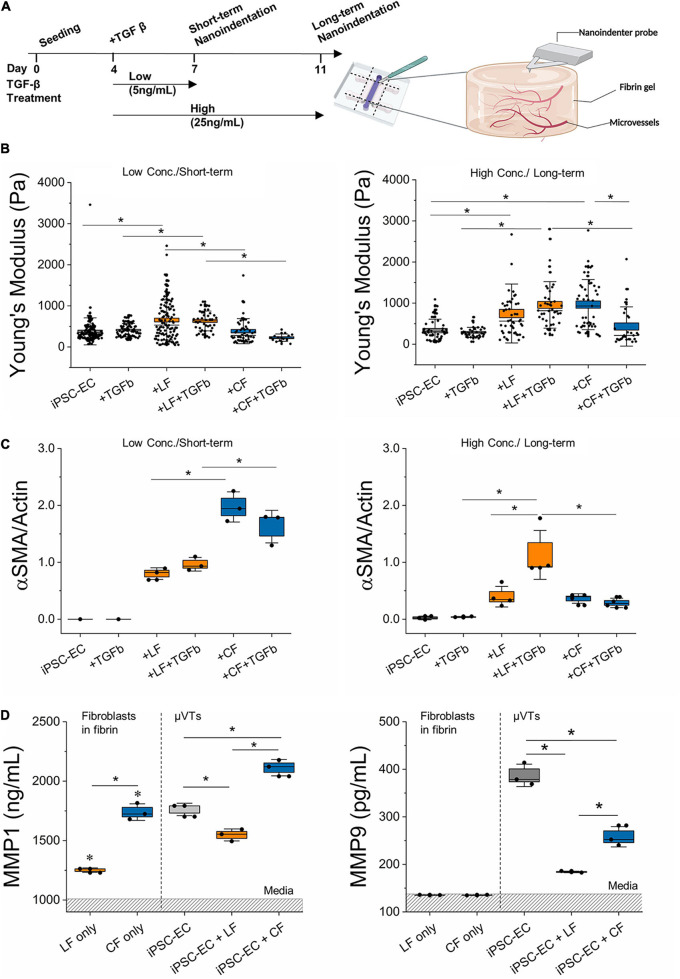
TGF-β treatment induces differential stromal-cell matrix remodeling. **(A)** Schematic diagram of TGF-β treatment regimens and subsequent nanoindentation experiments. **(B)** Measured effective Young’s modulus of the microvessels for the low concentration/short-term and high concentration/long-term conditions. **(C)** Relative αSMA expression in the different treatment regimens determined by Western Blot (for iPSC-EC low conc./short-term *n* = 3 samples were pooled). **(D)** MMP 1 and MMP 9 expressed in TGF-β treated microvascular tissues measured by ELISA. Dashed region is the mean value for media. Significance is shown by *P < 0.05, using a one-way ANOVA and a subsequent Tukey means comparison test. Box plots demonstrate SD (outer whiskers) and SE (box edge).

Transforming growth factor-β has been widely utilized in replicating fibrotic hallmarks, such as the activation of myofibroblasts and increased ECM protein deposition (Wynn and Ramalingam, 2012; Pardali et al., 2017; Lemos and Duffield, 2018; Sun et al., 2020). Here, microvascular tissues were treated on day 4 (after the formation of nascent microvessels) with either one of two different TGF-β treatment regimens: low concentration/short-term and high-concentration/long-term (Figure 4A). These regimens served as lower and upper TGF-β thresholds examined previously in alternative *in vitro* fibrotic models (Thannickal et al., 2003; Jeon et al., 2014; Walker et al., 2019; Mastikhina et al., 2020; Mejías et al., 2020). Following treatment in the μVTs, we measured changes in stiffness using nanoindentation (Figure 4B) and corresponding αSMA (Figure 4C) and collagen levels (Supplementary Figure 5) by western blotting. In both regimens, there were no changes in the measured stiffness, αSMA expression, or collagen I expression between the treated and untreated hiPSC-EC microvessels. On the other hand, TGF-β treated LF co-cultures significantly increased in stiffness and correspondingly increased in αSMA expression. Surprisingly, TGF-β treated cardiac-like μVTs resulted in a decrease in the measured stiffness and αSMA expression; however, the microenvironment stiffness was only significantly affected under high-concentration/long-term treatments. No measurable differences in collagen expression were observed between the control and treated groups.

In an attempt to correlate the observed changes in stiffness with changes in ECM protein degradation, we next measured MMP activity in response to TGF-β treatment in the various μVTs (Figure 4D). Supernatants were collected from μVTs treated by the short-term/low concentration regimens and were analyzed by ELISA. Fibroblast-only controls (in fibrin) showed comparable expression to growth media for MMP-9, where FBS contains MMPs, as previously observed (Hu and Beeton, 2010). There was a marked increase in the endogenous expression of MMP 1 and -9 in vascularized μVTs. MMP-1 was highly expressed in the cardiac co-culture, in contrast with the low expression in the lung co-culture. Fibroblast only cultures are significantly different from their respective μVTs. MMP-9 was expressed in higher amounts in the treated hiPSC-EC μVTs than in both co-cultures, corresponding with their relatively soft microenvironment, particularly in comparison to lung μVTs.

Phase-contrast images taken on days 7 and 10 also showed significant morphological changes resultant from the high-concentration/long-term regimen (Supplementary Figure 4). In general, TGF-β treated microvessels appear thinner than the untreated controls; vascular density decreased as the lumens narrowed and were more occluded. Overall, fibroblast co-cultured microvessels were more resilient to the treatment compared to the monoculture hiPSC-EC vessels.

## Discussion

**FIGURE 5:**
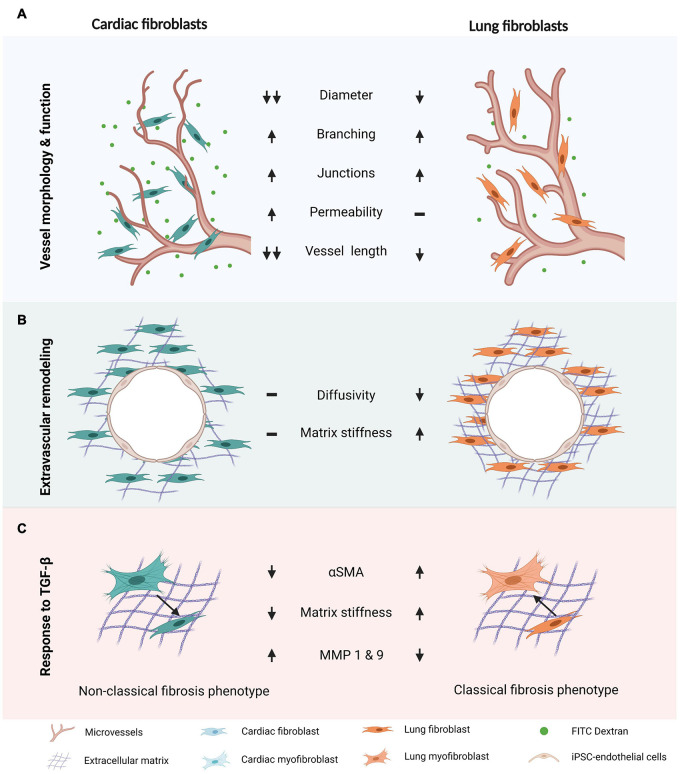
Differential response of cardiac and pulmonary-like microvascular tissues in induced fibrosis. **(A)** Vessel morphology and endothelial barrier function are impacted by stromal cell type. **(B)** Extravascular matrix is differentially remodeled in cardiac and lung microvascular tissues. **(C)** There is a differential response to TGF-β stimulation in the cardiac and lung microvascular tissues. In **(A,B)**, trends are in comparison to the monoculture microvascular tissue properties.

Leveraging our experience in generating perfusable microvascular networks (Haase et al., 2019, 2020), we established perfusable lung- and cardiac-like microvascular tissues on-chip. To the best of our knowledge, we are the first to report microvascular morphometric changes in hiPSC-EC derived microvessels and alterations in extravascular matrix properties, due to the presence of tissue-specific fibroblasts. We employed hiPSC-ECs to understand how tissue-dependent stromal-endothelial interactions influence the fibrotic response in a controlled manner. This approach allowed us to isolate the influences of the local stromal population and minimize tissue-dependent endothelial cell heterogeneity, which has been shown to influence health and disease (Aird, 2007; Augustin and Koh, 2017; Pasut et al., 2021). Our results demonstrate a clear impact of lung and cardiac fibroblasts on the formation of hiPSC-EC derived microvessels. In both co-cultures, fibroblasts clearly associated with vasculature and strongly impacted their morphology, largely resulting in smaller diameter vessels and increased branching (Figure 1B and Supplementary Figure 2). Other *in vitro* models have demonstrated the importance of stromal-endothelial cell crosstalk in promoting vessel stability (Whisler et al., 2014; Zeinali et al., 2018; Mejías et al., 2020). Despite the same initial seeding ratio, cardiac fibroblasts, unlike those from the lung, led to narrower vessels and reduced endothelial barrier function. Moreover, LFs, as opposed to cardiac, significantly reduce extravascular diffusivity and increased the overall stiffness of μVTs in comparison to hiPSC-EC μVTs. Endothelial-stromal cell crosstalk results in varied effects for lung and cardiac μVTs and their extravascular microenvironments, which is summarized in Figure 5.

Considering its strong association with fibrosis, TGF-β was used to induce an increased fibrotic-like state in our μVTs. Treatment with TGF-β had a severe impact on vascular stability, resulting in loss of viable networks in the absence of stromal cells (Supplementary Figure 4). Interestingly, this effect was abrogated by the presence of lung and cardiac fibroblasts. In all TGF-β treated microvessels, network density appeared reduced, similar to previous observations *in vitro* in HUVEC-derived microvessels and bone marrow-derived human mesenchymal stem cells (Jeon et al., 2014). Reduction in microvessel density has been implicated in fibrosis *in vivo*, but whether rarefaction is an initiator, contributor, or a consequence of fibrosis is still unknown (Sun et al., 2020). A lower vascular density can advance fibrosis via hypoxia-induced fibroblast activation (Ballermann and Obeidat, 2014) and the upregulation of LOXL2 production (a prominent actor in collagen crosslinking) in exosomes secreted from hypoxic endothelial cells (de Jong et al., 2016). Future studies on vascular rarefaction in the context of TGF-β induced fibrosis on-chip could help elucidate its role in fibrosis.

Fibrotic mechanisms in cardiac and lung fibrosis have been hypothesized to differ in terms of apoptotic cell sources, outcome, timeline, collagen degradation, and anatomic location (Johnson and DiPietro, 2013; Murtha et al., 2017). A possible contributor to these observed differences could be attributed to the differential fibroblast response in vascular-matrix remodeling marked by differences in expressed αSMA levels, inflammatory profiles, MMP activity, and associated microvascular tissue stiffness, as observed herein. TGF-β treatment resulted in concentration-and-time-dependent changes in extravascular matrix remodeling for the lung- and cardiac-like μVTs (Figure 4B). For the lung-like μVTs, an increase in tissue stiffness and a corresponding significant increase in αSMA was observed due to long-term treatments at high concentrations (Figures 4B,C and Supplementary Figure 5). Various groups have reported different concentrations (1–50 ng/ml) of TGF-β and treatment durations (24 h to 3 weeks) to induce fibrosis *in vitro*; longer treatment periods have been observed to induce distinct classical fibrotic phenotypes with human donor cells (Thannickal et al., 2003; Jeon et al., 2014; Walker et al., 2019; Mastikhina et al., 2020; Mejías et al., 2020). However, the opposite trend was observed here in the cardiac vessels – reduced stiffness and a corresponding lower expression of αSMA (Figure 4). Notably, untreated cardiac-like μVTs resulted in a significant increase in stiffness over the long-term culture period, yet the addition of TGF-β led to a softening effect. Tissue stiffness has been reported previously to play a role in fibroblast activation and tissue remodeling (Shi et al., 2013; El-Mohri et al., 2017; Yeh et al., 2017; Notari et al., 2018; Wang et al., 2020, 2021). Softer microenvironments reduce fibroblast activation *in vivo* and in cardiac explant models (Notari et al., 2018; Wang et al., 2021). This decreased stiffness from TGF-β treatment could contribute to the reduced αSMA expression – taken here as an indicator of a reduced activated fibroblast population.

It has been suggested that the abundance of inflammatory factors in the early stages of cardiac fibrosis reduces TGF-β responsiveness, delaying myofibroblast activation and ECM protein deposition (Dobaczewski et al., 2011; Frangogiannis, 2014; Murtha et al., 2017). Our hiPSC-EC derived vessels (with and without stromal cells) express inflammatory cytokines (Figure 3C). Given that our vessels are cultured in a fibrin hydrogel, it is also possible that the normal (untreated) microvessel environment on-chip promotes a wound-healing state. Increased ENA-78 and IL-6 expression in these vessels suggest an inflammatory-like environment, and both factors have been previously implicated in fibrosis (Walz et al., 1997; Arenberg et al., 1998; Wynn and Ramalingam, 2012; Tanaka et al., 2014). Moreover, proinflammatory cytokines have been implicated in endothelial junction disruption, influencing endothelial barrier function (Wallez and Huber, 2008; Dejana et al., 2009; Alimperti et al., 2017). μVTs showed increased expression of inflammatory factors in both the cardiac fibroblast-only and cardiac-microvessel co-culture (Figure 3C). Additionally, a 2D array showed increased expression of inflammatory factors from cardiac, in comparison to LFs despite both being cultured in low-serum (2% FBS) medium (Supplementary Figure 3A). From our results, we expect that the differences in inflammatory signaling (increased IL-6) contribute to the observed differences in endothelial barrier function in the cardiac co-culture system (Figures 2B,C and Supplementary Figure 3A), but this needs to be confirmed in future studies. This increased inflammatory phenotype of the cardiac fibroblasts is consistent with reports of a prolonged inflammatory stage in cardiac fibrosis (Murtha et al., 2017). These differences in pro-inflammatory cytokines could delay the response to TGF-β in the cardiac-like μVTs, contributing to the different timelines observed in cardiac and pulmonary fibrosis.

The complex interplay between tissue inhibitors of metalloproteases (TIMPs) and MMPs also regulate the fate of ECM remodeling; however, there is limited information on the role of TIMPs in lung fibrosis (Murtha et al., 2017). Moreover, lung and cardiac fibrosis both lead to increased collagen synthesis; yet, cardiac fibrosis has been shown to result in increased collagen degradation during early stages of the disease. Increased remodeling activity results in the breakdown of the collagen network in the case of cardiac fibrosis, while in the lung, it leads to collagen processing and maturation (Fan et al., 2012; Johnson and DiPietro, 2013; Murtha et al., 2017). Additionally, the upregulation of protease inhibitors, such as TIMPs, occurs after the inflammatory stage in cardiac fibrosis further contributing to the reported characteristics of early-stage fibrosis. These differences could explain the contrasting trends between cardiac- and lung-specific μVTs, which quite clearly follow unique timelines in ECM remodeling and production. Additionally, the differential effects of TGF-β on matrix regulation via MMP regulation could contribute to these differences (Uría et al., 1998; Padua and Massagué, 2009; Lian et al., 2021). TGF-β has been reported to up-regulate MMPs and down-regulate TIMPs in cancer cells, human fibroblasts, and endothelial cells (Uría et al., 1998; Padua and Massagué, 2009; Hsieh et al., 2010; Moore-Smith et al., 2017); conversely, it has also been implicated in downregulation of MMPs and the upregulation of TIMPs (Leivonen et al., 2013), suggesting a tissue and context-dependent role in matrix remodeling (Padua and Massagué, 2009; Krstic and Santibanez, 2014).

Our results demonstrate MMP-1 and MMP-9 (which act on collagen I and collagen IV, respectively) activity in all TGF-β stimulated μVTs (Figure 4D). Although FBS in the culture medium contains MMPs (Hu and Beeton, 2010), there was a marked increase in endogenous expression of MMP- 1 and -9 in treated microtissues. MMP-1 is an interstitial collagenase and acts on collagen I-III, VII, VIII, and gelatin. MMP-1 was highly expressed in the cardiac co-culture and lowest in the lung co-culture. Collagen I is the most abundant structural protein in fibrotic remodeling, and increased degradation activity could partially explain the decreased tissue mechanical properties observed in the TGF-β treated cardiac μVTs (Figure 4). MMP-9, which acts on collagen IV (a basement membrane protein), was also differentially expressed in the two tissues. It is naturally lower in fibroblasts and is mostly expressed by endothelial cells, to modulate their basement membrane (Lindner et al., 2012; Florence et al., 2017; Quintero-Fabián et al., 2019). MMP-9 was expressed highly in hiPSC-EC μVTs, suggesting that endothelial cells are largely responsible for MMP-9 induced remodeling. Lower MMP-1 activity in the lung co-cultures could explain a reduced ECM degradation and increased apparent stiffness of these tissues.

The differential expression of MMPs in tissue-specific fibroblasts has been explored by other groups (Collins et al., 2001; Lindner et al., 2012). Lindner et al. explored the mRNA expression of a range of MMPs in response to inflammatory cues using tissue necrotic factor-alpha (TNF-α) treatment on 2D cultured human fibroblasts. Cardiac fibroblasts, compared to LFs, showed a higher expression of MMP1. Conversely, they report that LFs showed a higher expression of MMP-9 than cardiac fibroblasts. While our data indicate that stromal cells alone in 3D fibrin gels do not express MMP-9 (above values seen in culture media), the mechanical cues from our 3D microenvironment and TGF-β treatment (as opposed to TNF-α) could contribute to these differences. Exploring the role of TIMPs, MMPs, the extent of collagen crosslinking (Tzortzaki et al., 2006; Barry-Hamilton et al., 2010), and cellular proliferation, in these tissue-specific microenvironments could also provide a better understanding of the different modes of vascular and extravascular remodeling.

Our data suggests the capacity of our model to recapitulate certain hallmarks of early-stage fibrosis (<2 weeks). Longer-term culture (21 days or more) might allow for the role of the endothelium in end-stage fibrosis to be revealed, where collagen accumulation is more evident. It has been suggested that the genetic modification of the ETV2 gene (to generate endothelial cells from fibroblasts) or the immortalization of endothelial lines using human telomerase reverse transcriptase can help extend the longevity of endothelial cells *in vitro* (Pham et al., 2018; Wan et al., 2021; Zhang et al., 2021)—which is a current limitation of our model and a major goal of our future studies. Beyond expected differences between various tissue sources, there is also expected variability between donor sources. Here, using primary cell lines we observed baseline differences in inflammatory cytokine expression and αSMA expression in stromal populations before TGF-β treatments (Supplementary Figure 3). This baseline αSMA expression in stromal cells could be, in part, attributed to the non-physiologic stiffness of the culture substrate. e.g., 3GPa for standard culture plastic vs 21 kPa for a healthy adult heart. Approaches toward “deactivating” or reducing the myofibroblast state *in vitro* are currently under investigation (Tatullo et al., 2016; Herum et al., 2017; Gilles et al., 2020; Mastikhina et al., 2020; Wang et al., 2021). However, our findings suggest that both the cardiac and LFs conserved their respective phenotypes, correlating with existing literature on differential fibrosis timelines, inflammatory profiles, and MMP activity. Future studies aim to derive all cell types from the same hiPSC donor which will provide valuable insight into patient-specific responses (from healthy and diseased sources). The pathogenesis of fibrosis in these two distinct organs will undoubtedly lead to differences in response times and remodeling behaviors, the exact mechanisms of which remain an open question and the focus of our future investigations.

In conclusion, functionally perfusable microvessels in cardiac- and lung-like microenvironments were developed on-chip to elucidate key stromal-endothelial interactions in vascular and extravascular tissue remodeling. Endothelial barrier function, vascular morphology, and matrix properties (tissue stiffness and diffusivity) are differentially impacted by the presence of lung and cardiac stromal cells. Classical hallmarks of fibrotic phenotypes were demonstrated in lung-like microenvironments, resulting in increased stiffness and αSMA expression in response to TGF-β treatment. Cardiac microvessels resulted in tissue softening upon TGF-β treatment and a correlated decrease in αSMA expression, suggesting tissue-specific differential matrix remodeling in comparison to lung μVTs. Differences in baseline pro-inflammatory cytokines, as well as MMP-1 and MMP-9 activity, between the two tissue type fibroblasts, correlate with the observed tissue-specific mechanisms seen *in vivo.* Our results lay the groundwork for future long-term studies into the mechanisms behind these varied fibrotic phenotypes observed and provide insight into tissue-dependent fibrotic pathogenesis. Given the sensitivity of the system, hiPSCs from patients suffering from fibrosis could be used in a similar approach in the future to examine potential therapeutics.

## Data Availability Statement

The raw data supporting the conclusions of this article will be made available by the authors at request, without undue reservation.

## Author Contributions

AA performed and analyzed experiments and wrote the manuscript. VB-S performed and analyzed western blot experiments and contributed to experimental planning. MC performed some ELISA experiments and contributed to experimental planning. RV, DC, and CH performed preliminary experiments. KH directed and performed some of the experiments and contributed to editing the manuscript. All authors contributed to the article and approved the submitted version.

## Conflict of Interest

The authors declare that the research was conducted in the absence of any commercial or financial relationships that could be construed as a potential conflict of interest. The handling editor MH shares the secondary affiliation of the authors AA and DC. All parties confirm the absence of any collaboration during review.

## Publisher’s Note

All claims expressed in this article are solely those of the authors and do not necessarily represent those of their affiliated organizations, or those of the publisher, the editors and the reviewers. Any product that may be evaluated in this article, or claim that may be made by its manufacturer, is not guaranteed or endorsed by the publisher.
