# Supplementary material for: Classical and Non-classical Fibrosis Phenotypes Are Revealed by Lung and Cardiac Like Microvascular Tissues On-Chip
Source: Front Physiol. 2021 Oct 6;12:735915. doi: 10.3389/fphys.2021.735915 (PMC8528192; doi:10.3389/fphys.2021.735915)
Supplement: Supplementary file 1 [file Data_Sheet_1.PDF]

Supplementary material

Classical and non-classical fibrosis phenotypes are revealed by lung and cardiac like microvascular tissues on-chip

Akinola Akinbote[1,2], Violeta Beltran Sastre[1], Marta Cherubini[1], Roberta Visone[3,4], Cynthia Hajal[4], Defne Cobanaglu[1,2], Kristina Haase[1]*

[1]European Molecular Biology Laboratory, Barcelona, Spain

[2]Heidelberg University, Heidelberg, Germany

[3]Politecnico di Milano, Milan, Italy

[4]Massachusetts Institute of Technology, Cambridge, USA

*Correspondence:

Kristina Haase

kristina.haase@embl.es

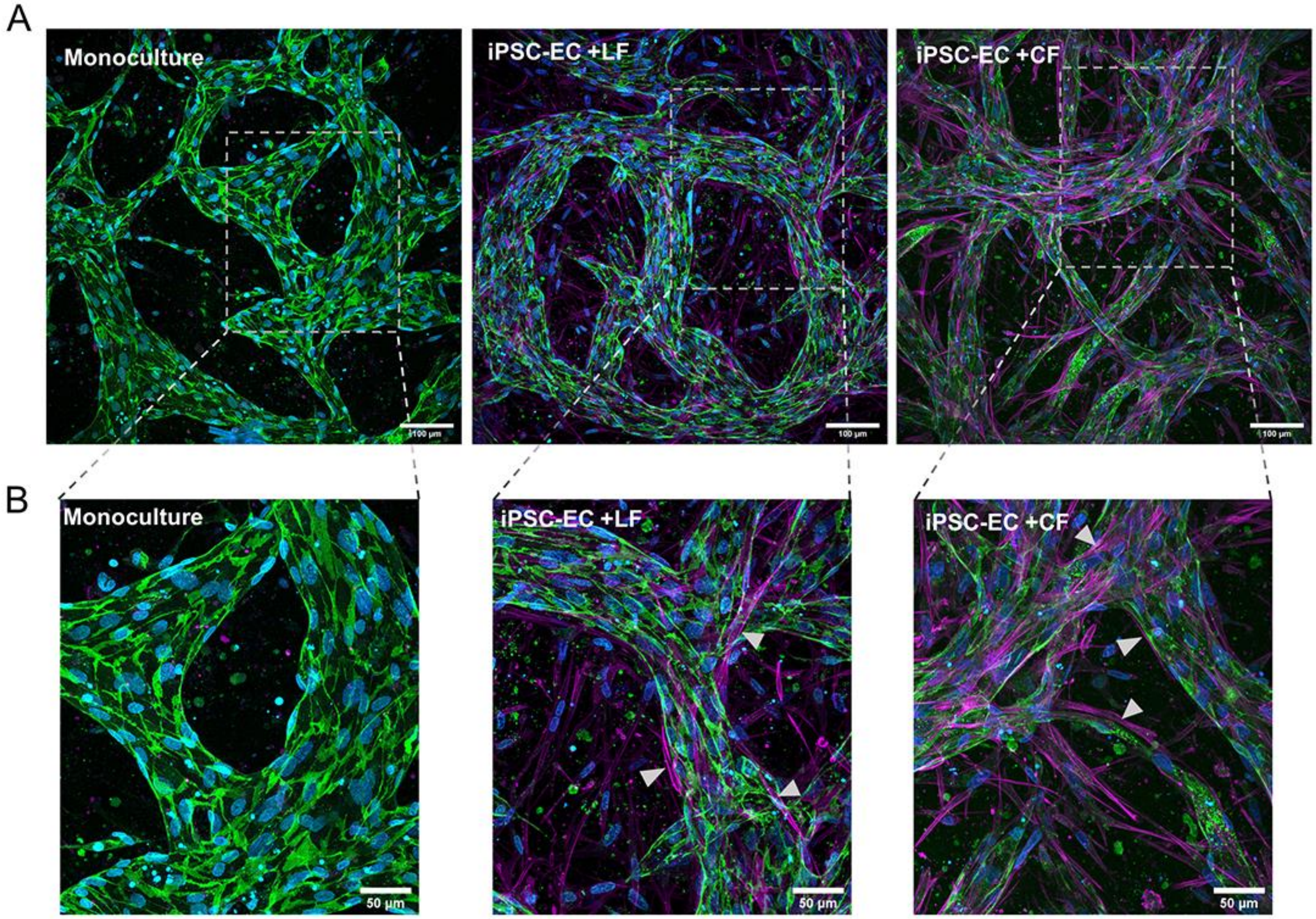

Supplementary Figure 1. Stromal cells impact the morphology of iPSC-EC derived microvessels. L-R: Microvessels derived from iPSC-EC monoculture, iPSC-EC and lung fibroblast co-culture, and hiPSC-EC and cardiac fibroblast co-culture. Microvessels were stained for CD31 (green) and counterstained with Dapi (blue) and phallodin (magenta). (A) Representative confocal images of microvascular networks taken at 20x showing stromal cell association with microvessels. Scale bar is 100µm. (B) Arrows inidcate stromal cell interaction with microvessels at an increased magnification. Scale bar is 50µm.

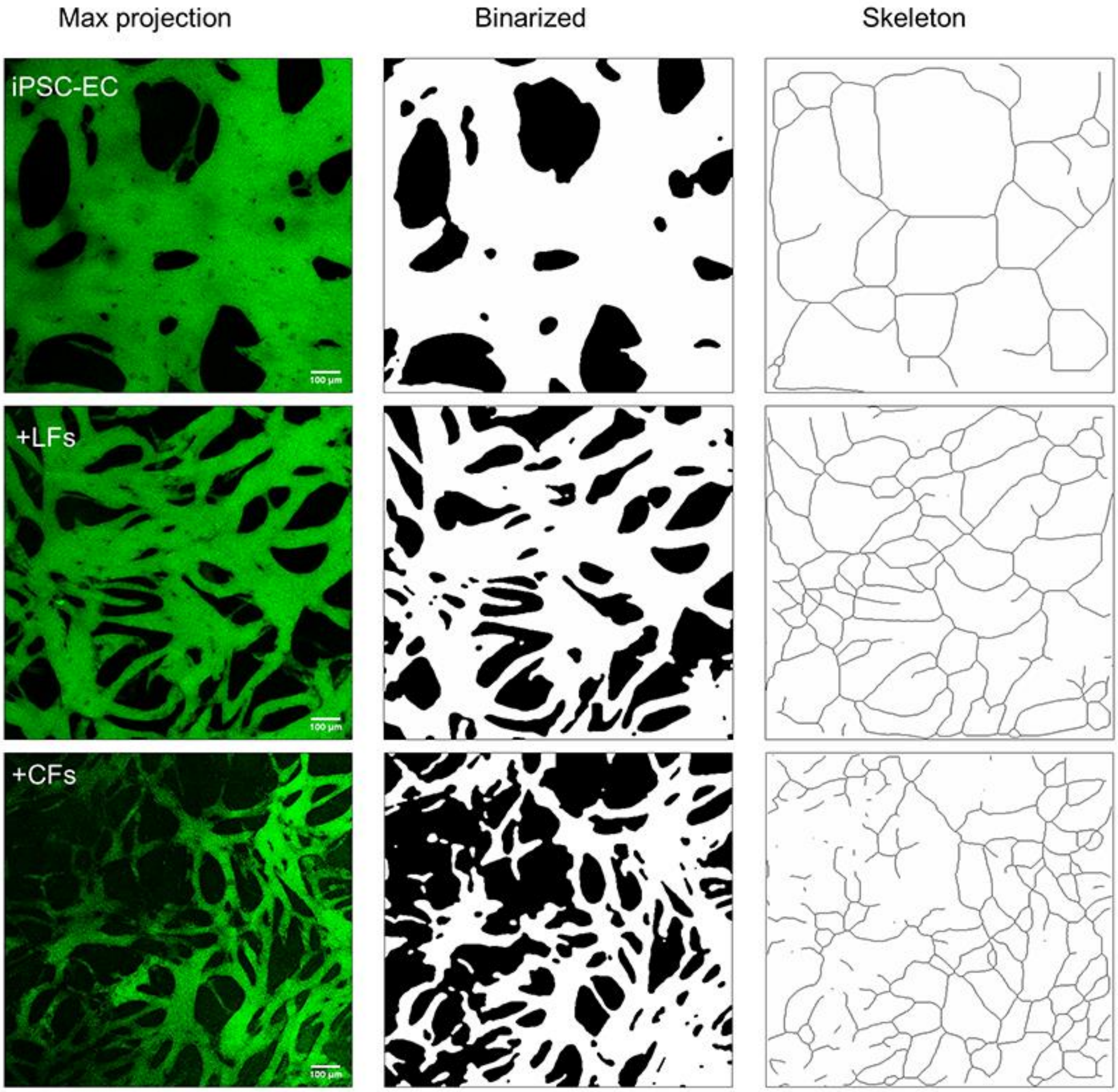

Supplementary Figure 2. Image segmentation pipeline. Representative images of perfused microvessels used in image processing for morphometric quantification. L-R: Max projection images of the FITC channel (green), binarized images showing the vessel area and the skeletonized image used to analyze network morphology. Scale bar is 100µm.

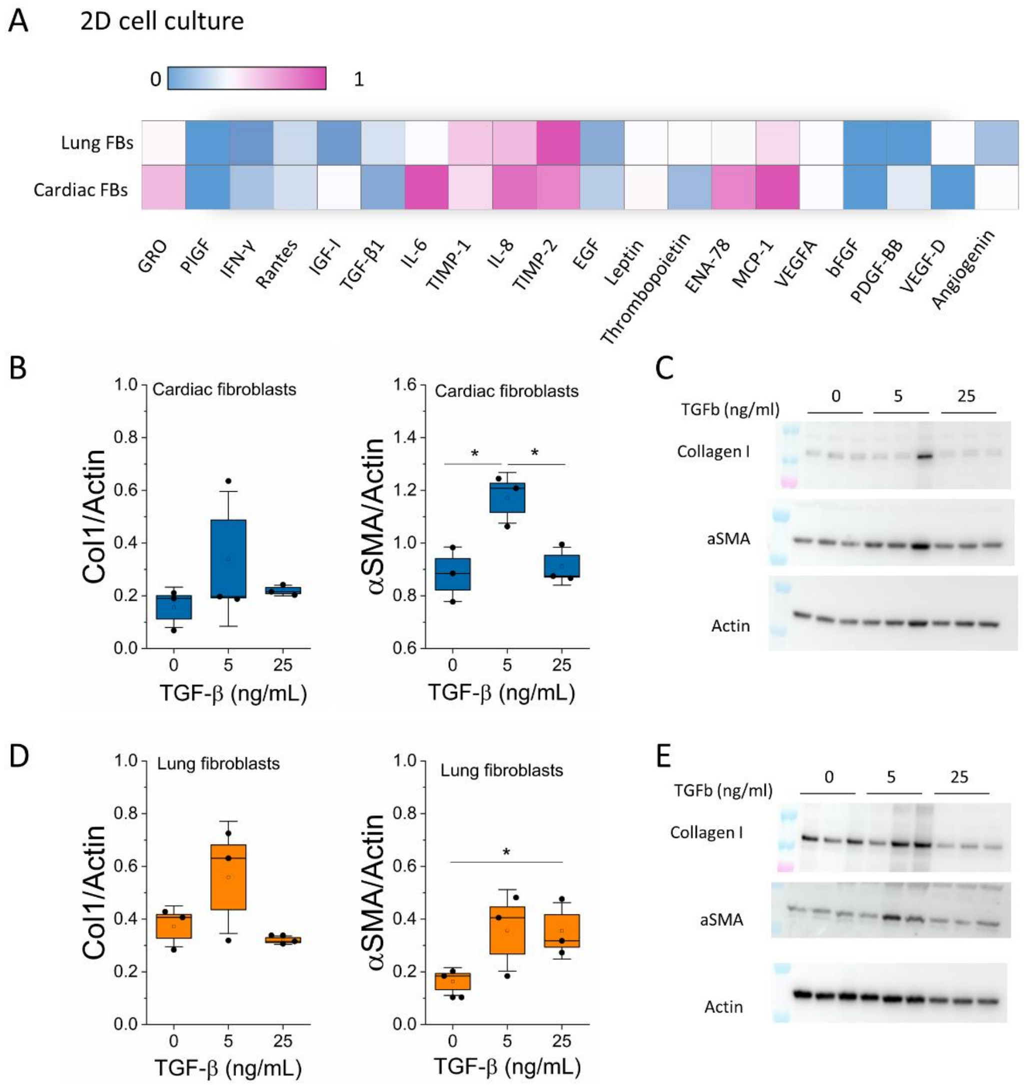

Supplementary Figure 3. Stromal cells in 2D show different baseline inflammatory cytokine profiles and a dose-dependent TGF-β response. (A) Heatmap showing relative baseline cytokine expression of fibroblasts cultured in 2D, quantified by a human angiogenesis array. (B) Collagen I and αSMA expression of TGF-β treated 2D cardiac fibroblasts relative to the actin expression, quantified by western blotting. (C) Corresponding western blots for treated 2D cardiac fibroblasts. (D) Collagen I and αSMA expression of TGF-β treated 2D lung fibroblasts relative to the actin expression, quantified by western blotting. (E) Corresponding western blots for treated 2D lung fibroblasts.

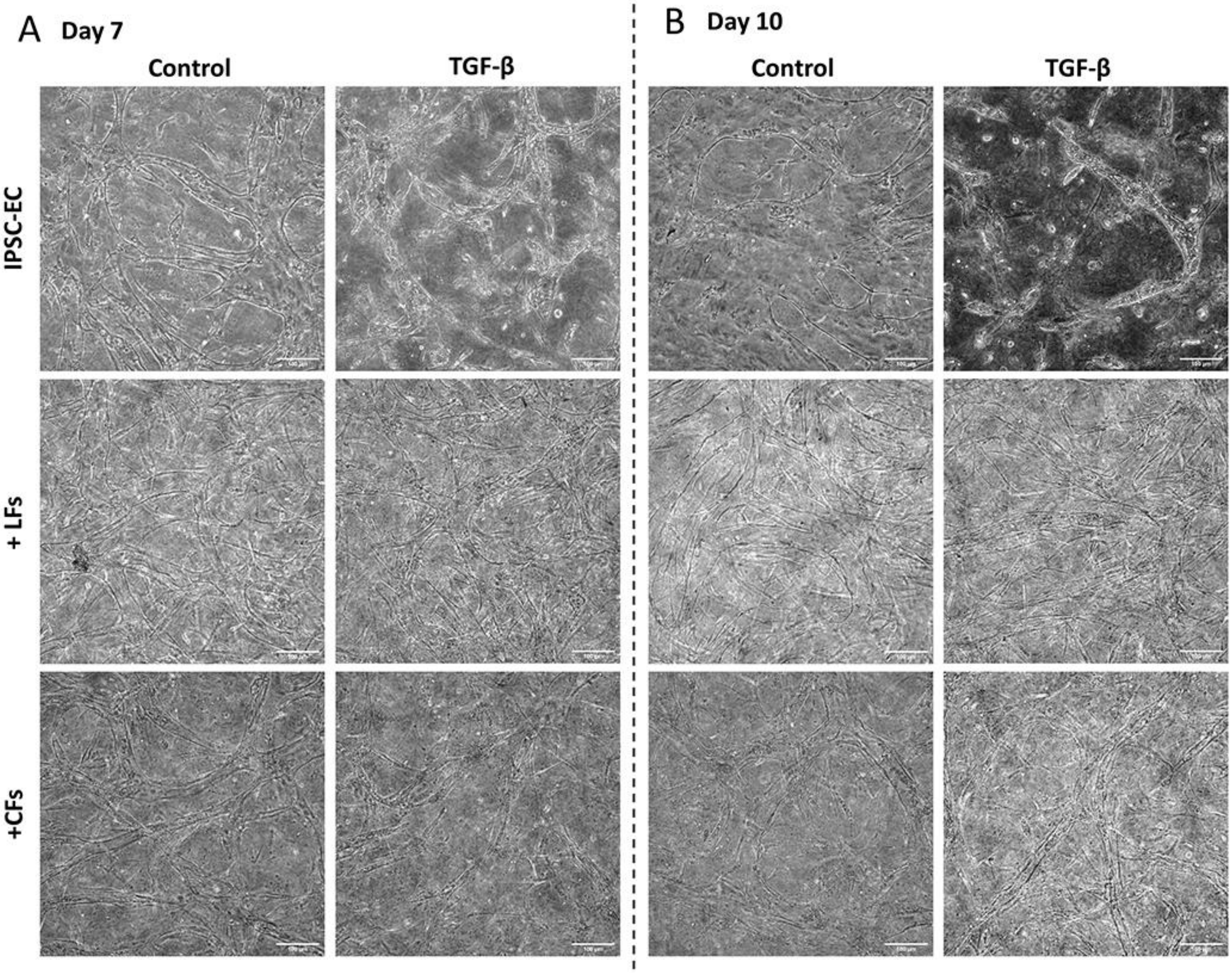

Supplementary Figure 4. TGF-β decreases microvascular network stability. Phase contrast images of microvessels treated with or without 25ng/ml TGF-β. (A) Representative images of microvessels taken at day 7. L-R: Treated vs untreated control. (B) Representative images of microvessels taken at day 10. L-R: Treated vs untreated control. Scale bars are 100µm.

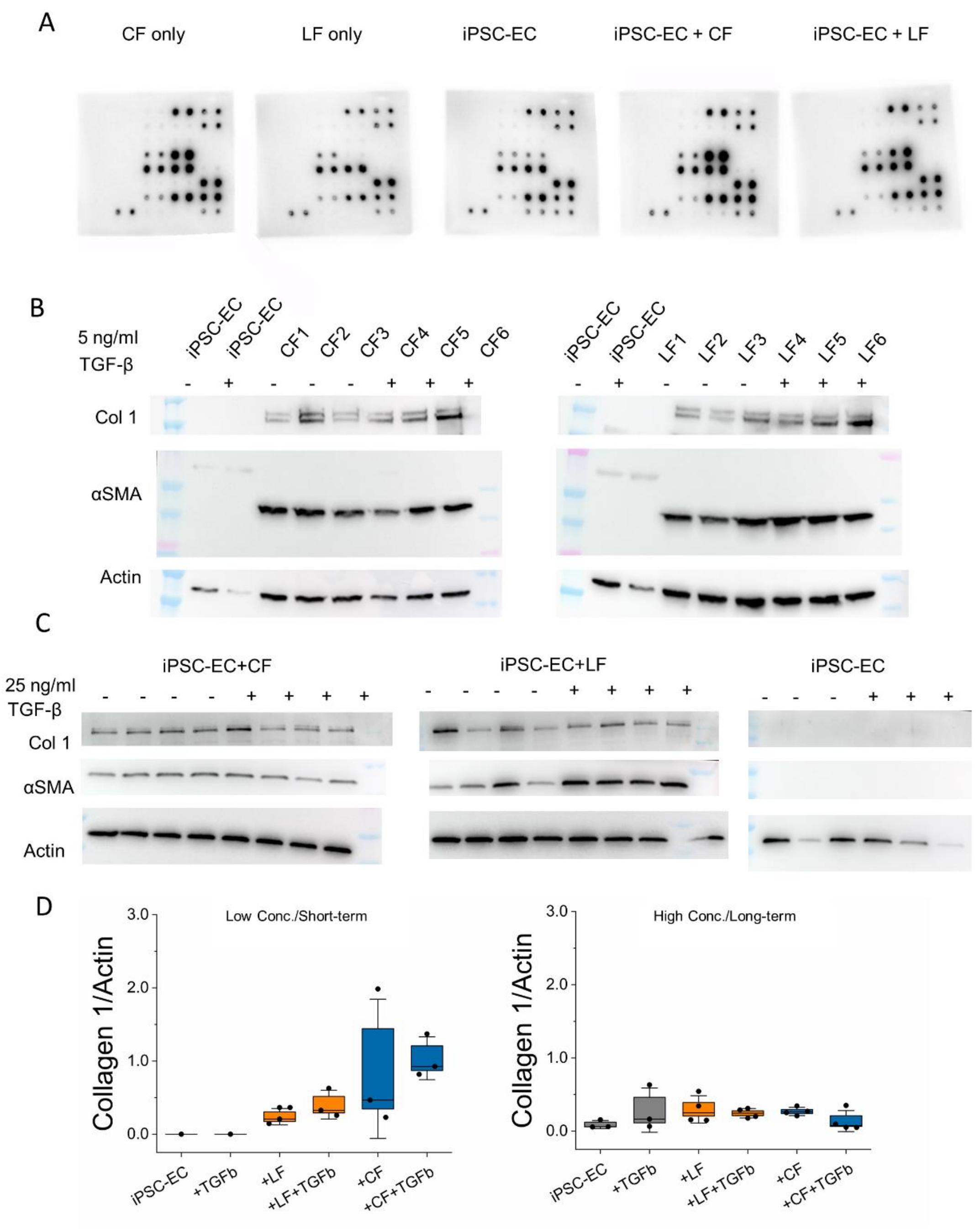

Supplementary Figure 5. Blots collected from 3D microvessels. (A) Angiogenesis array used to quantify relative cytokine expression at day 5. (B) Microvessels treated with low dose/ short-term TGF-β at 5ng/ml for 7 days. Collagen I and αSMA expression were quantified relative to the actin expression by western blotting. (C) Microvessels treated with high dose/ long-term TGF-β at 25ng/ml for 11 days. Collagen I and SMA expression were quantified relative to the actin expression by western blotting. D. Quantification of Collagen 1 in 3D microvessels from (Left) low and high (Right) concentration treatments, corresponding with representative blots in B and C, respectively. There were no significant changes in normalized collagen I expression.

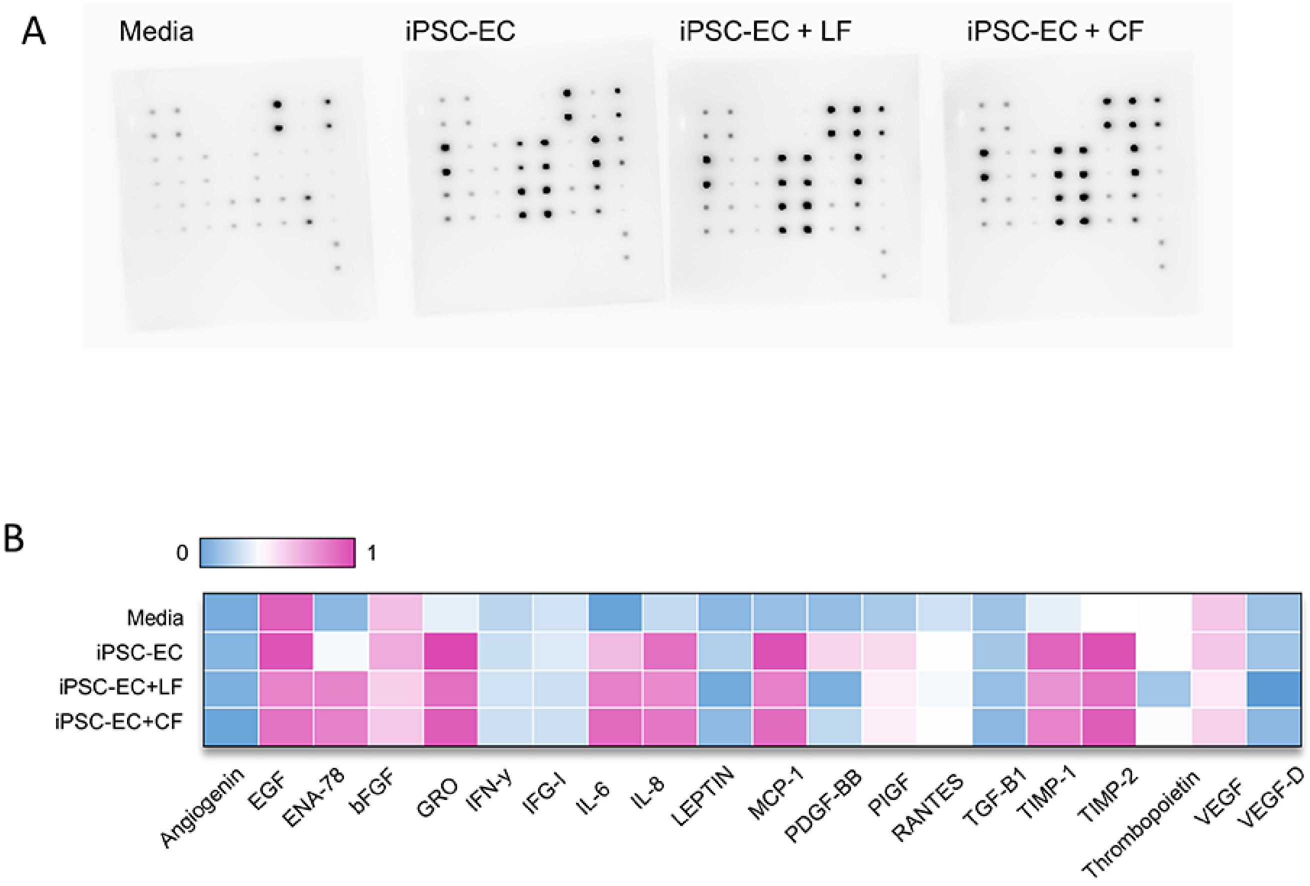

Supplementary Figure 6. Blots collected from 3D microvessels highlight cytokine expression in media and microvascular tissues. (A) Human angiogenesis array used to quantify relative cytokine expression at day 5. (B) Heatmap showing cytokine expression of 3D microvascular tissues cultured compared to growth media.
